# Multi-trait GWAS identifies pleiotropic loci associated with colorectal cancer in East Asian populations

**DOI:** 10.3389/fgene.2025.1590652

**Published:** 2025-04-15

**Authors:** Xiqi Chen, Yong Zhu, Peng Zhong, Guangdong Xie

**Affiliations:** ^1^ Department of Emergency Surgery, Affiliated Hospital of Shandong University of Traditional Chinese Medicine, Jinan, Shandong, China; ^2^ Department of Cardiology, Jining No.1 People’s Hospital, Jining, Shandong, China

**Keywords:** multi-trait GWAS, colorectal cancer, pleiotropic loci, East Asian populations, BioBank Japan

## Abstract

**Introduction:**

While genome-wide association studies (GWAS) have identified numerous susceptibility loci in Colorectal cancer (CRC), most findings are based on European populations. Additionally, CRC shares genetic architecture with other traits, and multi-trait analysis can improve the discovery of pleiotropic loci.

**Materials and methods:**

We conducted a multi-trait GWAS using the Multi-Trait Analysis of GWAS (MTAG) framework, leveraging large-scale genomic and phenotypic data from BioBank Japan (BBJ). We also examined genetic correlations between CRC and 70 complex traits, followed by local genetic correlation analysis and enrichment of heritability partitioned by chromatin states and tissue types.

**Results:**

We identified 25 genome-wide significant loci for CRC and colon polyps, including three novel loci in East Asian populations: *BET1L* (rs12226698, 11p15.5), *OAS1* (rs2525858, 12q24.13), and *BMP2* (rs4813802, 20p12.3). While *BMP2* had been previously reported in European CRC studies, *BET1L* and *OAS1* represent novel associations in East Asians. Colocalization analysis confirmed strong shared association signals between *BET1L* and *OAS1* in CRC and colon polyps, supporting their pleiotropic effects in colorectal neoplasia. *BET1L* was further identified in the multi-trait analysis of CRC and myocardial infarction. Similarly, *OAS1* was significantly associated with CRC and angina pectoris. Functional annotation revealed that these loci serve as expression quantitative trait loci (eQTLs) in colorectal tissues and immune-related pathways.

**Conclusion:**

Our study identifies novel pleiotropic loci associated with CRC in East Asians, emphasizing the importance of population-specific genetic studies. The findings provide new insights into the genetic architecture of CRC and its shared pathways with other complex diseases.

## 1 Introduction

Colorectal cancer (CRC) is one of the most common malignancies and a leading cause of cancer-related mortality worldwide (Lu et al., 2019; Pardamean et al., 2023). In East Asian countries, CRC incidence has risen sharply over the past few decades, making CRC a growing public health concern in this population. Rapid shifts in diet and lifestyle accompanying economic development have been implicated in this increasing CRC burden in East Asia (Pardamean et al., 2023). This epidemiological trend underscores the need to better understand all factors contributing to CRC risk in East Asian populations, including genetic predisposition.

Inherited genetic factors are known to play a significant role in CRC etiology (Lu et al., 2019). High-penetrance germline mutations in genes such as *APC* and DNA mismatch repair genes (e.g., *MLH1*, *MSH2*) cause familial CRC syndromes, but these rare mutations collectively account for less than 6% of all CRC cases (Lu et al., 2019). The majority of genetic susceptibility to CRC is polygenic. To date, genome-wide association studies (GWAS) have identified over 50 common loci associated with sporadic CRC risk, yet these known variants explain only a small fraction of the disease’s heritable risk. Notably, the proportion of “missing” heritability is even greater in populations of non-European ancestry (Lu et al., 2019), suggesting that many risk loci remain undiscovered in under-studied groups.

Population-specific genetic studies are therefore critical for elucidating CRC risk in East Asians. Differences in genetic architecture and environmental exposures between East Asian and Western populations mean that certain CRC susceptibility variants may be unique to East Asians or exhibit stronger effects in this group (Lu et al., 2019). Indeed, large GWAS efforts in East Asian cohorts have begun to reveal novel risk loci that were not detected in prior studies of European populations. For example, a recent consortium study of over 70,000 East Asian individuals identified 13 new CRC susceptibility loci (Lu et al., 2019), highlighting the value of ancestrally diverse research. These findings reinforce the importance of conducting GWAS in East Asian populations to capture the full spectrum of CRC genetic risk factors.

Another avenue to improve locus discovery is to leverage the pleiotropic nature of complex traits. Many genetic loci confer risk for multiple related phenotypes, a phenomenon known as pleiotropy. For instance, one of the first identified pleiotropic cancer loci is on chromosome 8q24, where certain variants are associated with susceptibility to CRC as well as breast, prostate, and ovarian cancers (Fehringer et al., 2016). Such cross-phenotype associations suggest that jointly analyzing correlated traits can expose shared genetic risk factors. Multi-trait GWAS approaches harness this strategy by testing for associations across multiple traits or disease phenotypes simultaneously. By leveraging the shared genetic architecture between related traits, multi-trait analyses can increase statistical power to detect risk loci that do not reach significance in single-trait GWAS (Fehringer et al., 2016). Indeed, recent studies have demonstrated that multi-trait or cross-phenotype analyses can uncover additional susceptibility loci that were missed by conventional univariate GWAS (Julienne et al., 2021; Wang et al., 2025; Ding et al., 2025; Song et al., 2024). This approach is particularly relevant for CRC, which shares genetic links with other traits (for example, inflammation, metabolic factors, or other cancers) that may jointly influence disease risk.

In this study, we performed a multi-trait GWAS in East Asian populations to identify pleiotropic genetic loci associated with colorectal cancer risk. Specifically, we leveraged large-scale genomic and phenotypic data from the BioBank Japan (BBJ), one of the largest East Asian biobanks, to investigate pleiotropic loci across multiple related traits. By integrating genetic associations from multiple phenotypes, our study aims to uncover population-specific risk variants and shed light on shared genetic mechanisms underlying CRC in this population. This work contributes to a more comprehensive understanding of CRC susceptibility in East Asians, with the potential to inform population-specific genetic risk assessment and prevention strategies.

## 2 Methods

### 2.1 GWAS data

GWAS summary statistics of studied traits from East Asians were downloaded from GWAS Catalog (Buniello et al., 2019), information of all included GWAS data from East Asians is summarized in Supplementary Table S1.

### 2.2 Global genetic correlation analysis

The global genetic correlation (*r*_*g*_) between each pair of colorectal cancer and studied traits was calculated using LD Score Regression (LDSC) and GWAS summary statistics is a widely used approach for quantifying genetic correlations between complex traits and diseases, offering valuable etiological insights and aiding in the prioritization of potential causal relationships. A key limitation of traditional methods for estimating genetic correlation from GWAS data lies in their reliance on individual genotype data, which is often unavailable, as well as the pervasive issue of sample overlap in meta-analyses. LDSC circumvents these challenges by employing cross-trait LD Score regression, a technique that depends solely on GWAS summary statistics and remains unaffected by sample overlap, ensuring robust and unbiased correlation estimates (Bulik-Sullivan et al., 2015). The formula used in LDSC is as follows:

$$E\left[\beta_{j}\gamma_{j}\right]=\frac{\sqrt{N_{1}N_{2}}r_{g}}{M}l+\frac{N_{s}r}{\sqrt{N_{1}N_{2}}}$$

where $\beta_{j}$ and $\gamma_{j\;}$ represent the effect sizes of ${SNP}_{j}$ on the two traits being tested, $N_{1}$ and $N_{2}$ are the sample sizes for the two traits, $N_{s}$ is the number of overlapping samples between the two traits, r is the phenotypic correlation in the overlapping samples, and, $l_{j}$ is the LD score. In this analysis, precomputed linkage disequilibrium scores for HapMap3 SNPs, derived from individuals of European ancestry in the 1000 Genomes Project (Bulik-Sullivan et al., 2015), were used. SNP markers with an imputation INFO score lower than 0.9 were excluded from the analysis (Bulik-Sullivan et al., 2015).

### 2.3 Cell-type-specific enrichment of SNP heritability

Research has shown that specific functional genomic categories play a disproportionate role in the heritability of complex diseases (Trynka et al., 2013). Stratified LD Score Regression (s-LDSC) was developed to quantify these contributions by partitioning heritability from GWAS summary statistics while considering linkage disequilibrium (LD). This method is computationally efficient and scalable to large datasets, making it ideal for genome-wide studies. Unlike traditional approaches that focus solely on genome-wide significant SNPs, s-LDSC leverages genome-wide information to provide a broader perspective on genetic architecture. By identifying cell type-specific elements and other functional genomic regions, it enhances understanding of the polygenic basis of complex traits and diseases, facilitating the prioritization of genomic regions for further functional studies (Finucane et al., 2015).

In this study, annotation data from six chromatin marks (DHS, H3K27ac, H3K36me3, H3K4me1, H3K4me3, and H3K9ac) across 88 cell types and tissues from the Roadmap project were used to partition SNP heritability across various traits. For each chromatin mark, cell type-specific annotations were categorized into nine groups: adipose, central nervous system, digestive system, cardiovascular, musculoskeletal/connective tissue, immune/blood, liver, pancreas, and others (Finucane et al., 2015). Annotation-specific enrichment values for each trait were visualized using a hierarchical clustering approach, with results represented on a color scale.

### 2.4 Local genetic correlation analysis

To complement the genome-wide genetic correlation estimates derived from LD Score Regression (LDSC), which integrates information from all genetic variants across the genome, we utilized ρ-HESS to assess local genetic correlations between trait pairs at a more refined level (Shi et al., 2017). Unlike LDSC, which provides an overall measure of shared genetic architecture, ρ-HESS allows for a region-specific evaluation of genetic covariance, offering deeper insights into the contributions of distinct genomic loci. ρ-HESS is specifically designed to quantify genetic correlation originating from variation within small genomic regions. This method relies solely on GWAS summary statistics and does not impose assumptions regarding the distribution of effect sizes of causal variants. Additionally, it accounts for both linkage disequilibrium (LD) structure and potential sample overlap between GWAS datasets, enhancing the reliability of the inferred local genetic relationships. The analytical process of ρ-HESS consists of several key steps. Initially, it determines the eigenvalues of LD matrices and computes the squared projections of GWAS effect size vectors onto the eigenvectors of these matrices for each trait. These projections are then used to estimate local SNP-heritability for each phenotype. Finally, based on the local heritability estimates, ρ-HESS derives local genetic covariance estimates along with their standard errors. In our study, all trait pairs that demonstrated significant genome-wide genetic correlation in the LDSC analysis (P < 0.01) were further examined using ρ-HESS. This approach enabled us to pinpoint specific genomic regions responsible for driving the global genetic correlation. To control for multiple testing, statistical significance was defined using a Bonferroni-corrected threshold of P < 0.05/n, where n (n = 1,703) represents the number of independent hypothesis tests conducted.

### 2.5 Multi-trait analysis of GWAS

The Multi-Trait Analysis of GWAS (MTAG) framework, a sophisticated meta-analysis approach, was utilized in this study to enhance the power of detecting trait-specific single nucleotide polymorphism (SNP) associations. Unlike traditional single-trait GWAS, which may overlook associations due to limited statistical power, MTAG integrates GWAS summary statistics across genetically correlated traits, leveraging their shared genetic architecture to improve locus discovery. This approach is particularly advantageous in studying complex diseases and polygenic traits, where overlapping genetic factors play a significant role. MTAG effectively addresses key challenges in multi-trait meta-analysis, including sample overlap and incomplete genetic correlation, issues that often lead to biases in conventional inverse-variance weighted meta-analysis. By incorporating information from related traits, MTAG provides a more refined genetic association signal while maintaining statistical robustness. The analysis begins with variant filtering, ensuring data quality by removing non-common SNPs, duplicated SNPs, and strand-ambiguous SNPs (Turley et al., 2018). Following this, MTAG estimates pairwise genetic correlations between traits using LDSC, which helps adjust the variance-covariance matrix of the random effect component (Bulik-Sullivan et al., 2015). This step ensures that the correlation structure among traits is properly accounted for, preventing inflation or deflation of association signals due to sample overlap or confounding. Subsequently, a random-effect meta-analysis is conducted to generate SNP-level summary statistics, improving the detection of shared genetic determinants across traits. In this study, we specifically focused on identifying pleiotropic variants with significant cross-trait effects. To prioritize SNPs with potential pleiotropic influence, we selected those reaching genome-wide significance (P < 5 × 10^−8^) in the MTAG analysis and exhibiting suggestive significance (P < 0.01) in the original single-trait GWAS. This stringent selection criterion ensured that only the most robustly associated SNPs were considered, increasing confidence in their potential biological relevance. The genomics inflation factor for the analysis of CRC and colon polyps, CRC and myocardial infarction, as well as CRC and angina pectoris were 1.057, 1.049 and 1.045 respectively, indicating minimal evidence of inflation.

## 3 Results

### 3.1 Genetic correlations between colorectal cancer and correlated traits

To investigate the shared genetic architecture of colorectal cancer with other complex traits in East Asian populations, we assessed genetic correlations across 70 phenotypes available in BBJ using LDSC. Among these, three traits exhibited significant genetic correlations with colorectal cancer (Table 1): angina pectoris (*r*_*g*_ = 0.41, SE = 0.078, P = 1.24E-07), myocardial infarction (*r*_*g*_ = 0.34, SE = 0.078, *P* = 1.56E-05), and colon polyp (*r*_*g*_ = 1, SE = 0.229, P = 3.86E-06).

**TABLE 1:** Genetic correlations between colorectal cancer and correlated traits.

Trait 1	Trait 2	*r*_*g*_	SE	*P*
Colorectal cancer	Angina pectoris	0.41	0.078	1.24 × 10^−7^
	Myocardial infarction	0.34	0.078	1.56 × 10^−5^
	Colon polyp	1	0.229	3.86 × 10^−6^

This table presents the genetic correlation (*r*_*g*_) estimates between colorectal cancer and three related traits: angina pectoris, myocardial infarction, and colon polyp. The genetic correlation (*r*_*g*_) was calculated using LDSC, with standard errors (SE) and *P*-values (*P*) provided for statistical significance. A higher *r*_*g*_ indicates a stronger genetic relationship between the traits.

The observed positive genetic correlations between colorectal cancer and cardiovascular conditions such as angina pectoris and myocardial infarction suggest potential shared genetic risk factors in East Asian populations. These findings align with previous research indicating links between metabolic and inflammatory pathways contributing to both colorectal cancer and cardiovascular diseases. The strong genetic correlation with colon polyps (*r*_*g*_ = 1) reinforces their well-established role as precancerous lesions in colorectal cancer development. Given the underrepresentation of East Asian populations in previous large-scale genetic studies, these results provide valuable insights into the genetic architecture of colorectal cancer in this population.

### 3.2 Partitioning heritability and chromatin marker enrichment in colorectal cancer and related traits

To further explore the genetic architecture of colorectal cancer and its correlated traits, we partitioned SNP heritability based on six chromatin markers and nine distinct cell types (Supplementary Figure S1). Our analysis revealed that colorectal cancer and colon polyps exhibited highly similar enrichment patterns across five chromatin markers, including H3K27ac, H3K4me3, H3K4me1, H3K36me3, and H3K9ac. Notably, when clustering the four phenotypes based on chromatin marker enrichment, colorectal cancer and colon polyps consistently grouped together, suggesting a shared epigenetic landscape between these conditions. This strong clustering further reinforces their well-established biological relationship, as colon polyps are recognized precursors of colorectal cancer. Indeed, both traits demonstrated specific genetic associations within various gastrointestinal tissues. For instance, H3K9ac and H3K36me3 markers were significantly enriched in Rectal Mucosa and Sigmoid Colon tissues for both colorectal cancer and colon polyps, highlighting a tissue-specific regulatory role in disease pathogenesis. Furthermore, in Small Intestine tissue, we observed significant enrichment of the DNase I hypersensitivity (DHS) marker across all four traits analyzed. This suggests that chromatin accessibility in this tissue may play a crucial role in the shared genetic basis of colorectal cancer and its related conditions. These findings provide compelling evidence that regulatory elements in gastrointestinal tissues contribute to the heritability of these traits and may underlie their pleiotropic effects, emphasizing the need for further functional validation of these epigenetic signals.

### 3.3 Local genetic correlation between colorectal cancer and related traits

**FIGURE 1:**
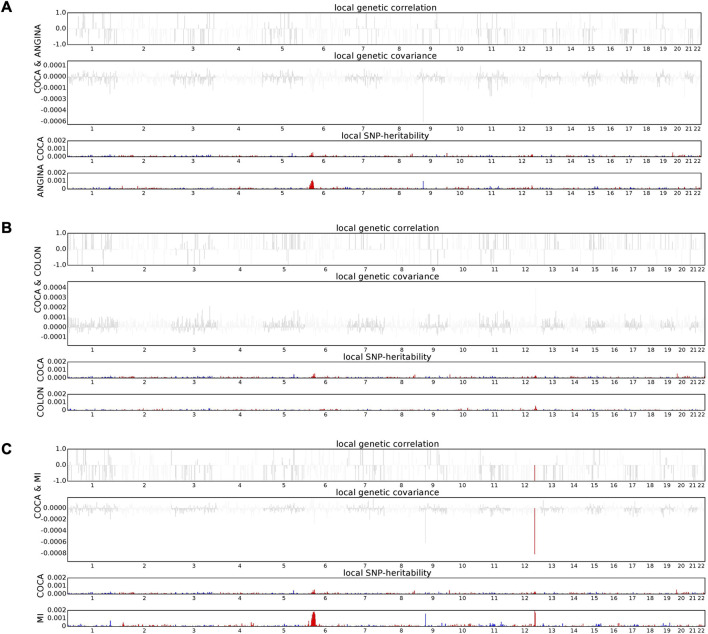
Local genetic correlation between colorectal cancer and related traits. **(A)** The Manhattan plot displays the estimates of local genetic correlation, genetic covariance, and SNP heritability between colorectal cancer and angina pectoris. **(B)** The Manhattan plot shows the estimates of local genetic correlation, genetic covariance, and SNP heritability between colorectal cancer and myocardial infarction. **(C)** The Manhattan plot presents the estimates of local genetic correlation, genetic covariance, and SNP heritability between colorectal cancer and colon polyps. A significant local genetic correlation was identified between colorectal cancer and myocardial infarction at the 12q24.11 locus, whereas no significant local correlation signals were observed for colorectal cancer with angina pectoris or colon polyps. Red bars indicate loci with significant local genetic correlation after multiple testing adjustment.

Building upon the observed genome-wide genetic correlations, we conducted a detailed local genetic correlation analysis to pinpoint specific genomic regions contributing to the shared heritability between colorectal cancer and related traits (Figure 1). Multiple testing corrections were applied to ensure the robustness of our findings. Interestingly, while colorectal cancer exhibited strong genome-wide genetic correlations with angina pectoris and colon polyps, no significant local correlation signals were detected for these traits, suggesting that their genetic relationships are likely driven by polygenic effects rather than specific genomic loci. However, a significant local genetic correlation was identified between colorectal cancer and myocardial infarction at the 12q24.11 locus. This region has been implicated in various complex diseases, suggesting a shared genetic architecture that may influence both colorectal cancer and myocardial infarction susceptibility. The 12q24.11 locus encompasses several genes, including *ATXN2*, *BRAP*, *SH2B3*, and *ALDH2*, which have been associated with multiple traits. For instance, *SH2B3* is known to play a role in immune system regulation, and its genetic variants have been linked to both cardiovascular diseases and autoimmune disorders. Similarly, *ALDH2* is involved in alcohol metabolism and has been associated with various health conditions, including cardiovascular diseases (Qiao et al., 2024).

### 3.4 Multi-trait GWAS analysis identifies pleiotropic loci for colorectal cancer and related traits

Given the evidence of shared genetic architecture between colorectal cancer (CRC) and genetically correlated traits, we performed a multi-trait GWAS using the MTAG framework to enhance the power of detecting pleiotropic loci that might have been missed in single-trait analyses. In this analysis of CRC and colon polyps, we identified 25 genome-wide significant loci (Supplementary Table S2), including three novel loci in East Asian populations: rs12226698 at 11p15.5 (*BET1L*), rs2525858 at 12q24.13 (*OAS1*), and rs4813802 at 20p12.3 (*BMP2*). While BMP2 has been previously reported in European populations, *BET1L* and *OAS1* represent newly identified risk loci in East Asians (Table 2).

**TABLE 2:** New pleiotropic loci identified in multi-trait GWAS of colorectal cancer and related traits.

SNP	CHR	BP	A1	A2	mtag_pval	Trait1	Trait2	pval1	pval2	beta1	beta2	mtag_beta	Gene
rs12226698	11	213,740	T	C	3.43E-09	Colorectal Cancer	Colon Polyps	8.19 × 10^−6^	6.23 × 10^−8^	−0.111	−0.173	−0.029	BET1L
rs2525858	12	113,030,906	G	A	9.14E-10	Colorectal Cancer	Colon Polyps	1.62 × 10^−6^	3.33 × 10^−7^	0.099	0.135	0.024	OAS1
rs73386631	11	202,017	T	C	8.05E-09	Colorectal Cancer	Myocardial Infarction	1.03 × 10^−6^	1.90 × 10^−6^	−0.112	0.097	−0.028	*BET1L*
rs2525858	12	113,030,906	G	A	9.02E-10	Colorectal Cancer	Angina Pectoris	1.62 × 10^−6^	4.60 × 10^−7^	0.099	−0.090	0.024	*OAS1*

This table shows significant SNPs, identified in the multi-trait GWAS (MTAG) analysis for colorectal cancer and related traits, including colon polyps, myocardial infarction, and angina pectoris. The columns include SNP ID, chromosome (CHR), base-pair position (BP), effect allele (A1), reference allele (A2), p-values from MTAG, and single-trait GWAS (pval1, pval2), effect sizes (beta1, beta2, mtag_beta), and the nearest gene.

**FIGURE 2:**
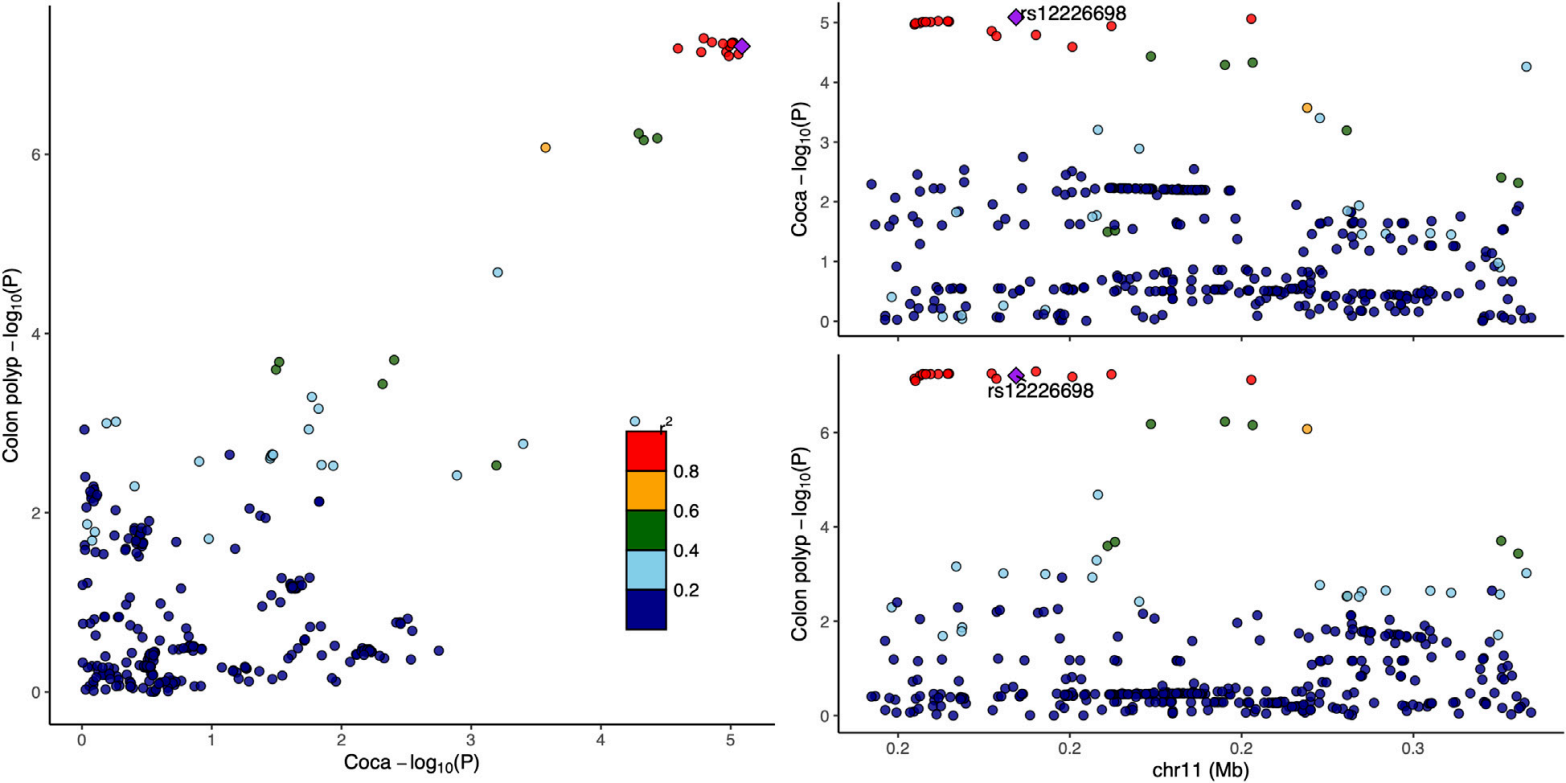
LocusCompare plot for rs12226698 in colorectal cancer and colon polyps. This figure This figure presents a LocusCompare plot for rs12226698, located at 11p15.5 (BET1L), illustrating the association signals between colorectal cancer and colon polyps. The x-axis represents the −log_10_ (*P*-value) for colorectal cancer, while the y-axis represents the −log_10_ (*P*-value) for colon polyps. Points are color-coded based on linkage disequilibrium (LD, r^2^) with rs12226698.

**FIGURE 3:**
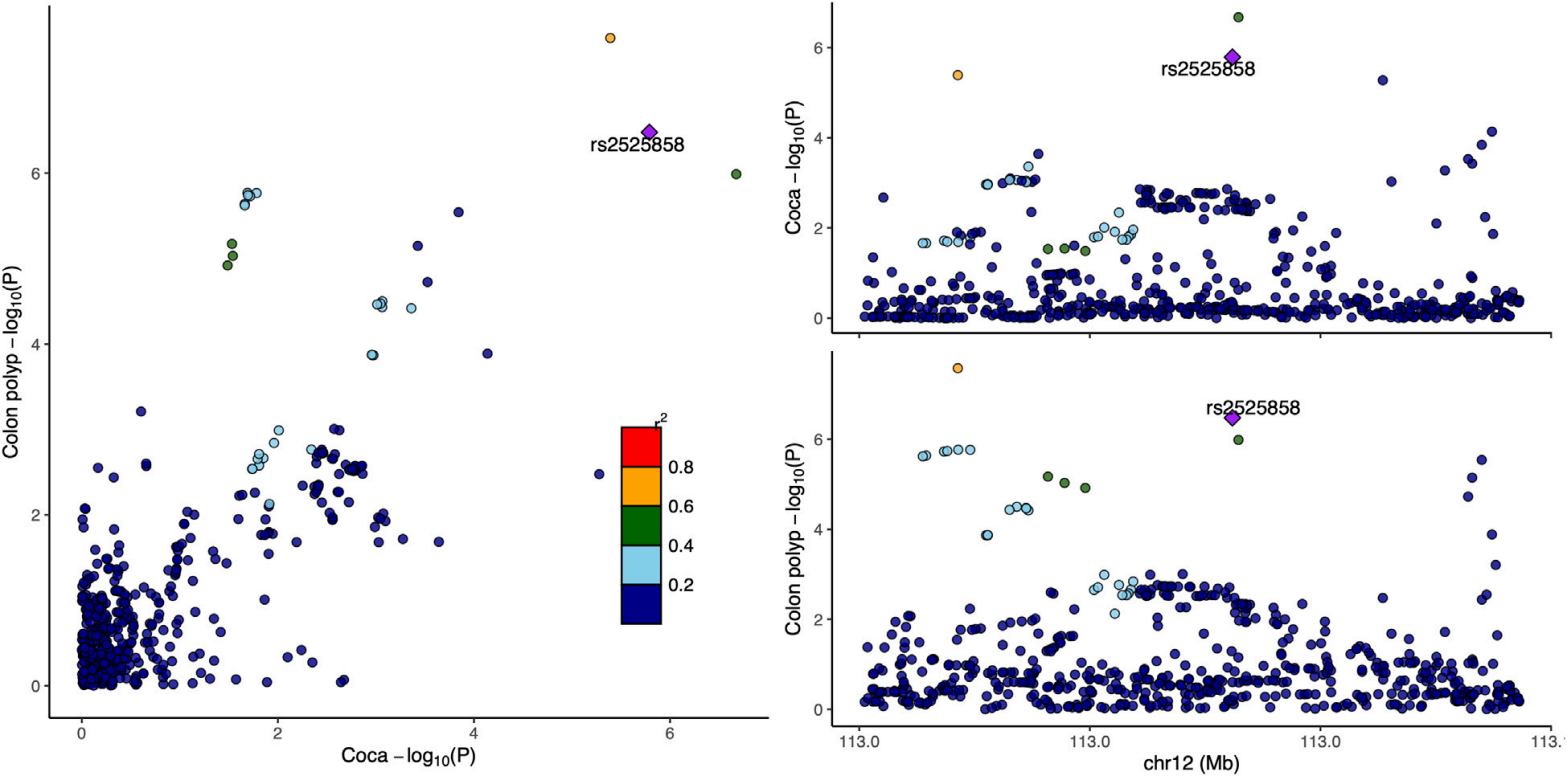
LocusCompare plot for rs2525858 in colorectal cancer and colon polyps. This figure presents a LocusCompare plot for rs2525858, located at 12q24.13 (OAS1), illustrating the association signals between colorectal cancer and colon polyps. The x-axis represents the −log_10_ (*P*-value) for colorectal cancer, while the y-axis represents the −log_10_ (*P*-value) for colon polyps. Points are color-coded based on linkage disequilibrium (LD, r^2^) with rs2525858.

Functional annotation using GTEx data revealed that rs12226698 is an eQTL for *BET1L* in multiple tissues (Supplementary Table S3), including the transverse and sigmoid colon, suggesting a potential regulatory role in colorectal tissue. *BET1L* (Bet1 Golgi Vesicular Membrane Trafficking Protein-Like) is involved in Golgi-mediated vesicular transport, playing a key role in intracellular trafficking and protein secretion, processes that are essential for maintaining epithelial homeostasis and may contribute to colorectal tumorigenesis. Notably, *BET1L* was also identified as a significant locus in the multi-trait analysis of CRC and myocardial infarction (Supplementary Table S4), suggesting that this gene may play a broader role in disease susceptibility through shared biological pathways, potentially involving cellular transport, metabolic regulation, or inflammatory signaling. To further investigate the pleiotropic effects of *BET1L*, we visualized the association signals for rs12226698 in both colorectal cancer and colon polyps using a LocusCompare plot (Figure 2). The strong correlation of association signals between these two traits at BET1L, coupled with high linkage disequilibrium (LD) and consistent effect directions, supports colocalization, indicating that the same causal variant may underlie risk for both traits. These findings reinforce the role of *BET1L* in the adenoma-carcinoma sequence, suggesting shared genetic mechanisms influencing both benign and malignant colorectal neoplasia. Similarly, rs2525858 is an eQTL for *OAS1* (Supplementary Table S3), a gene encoding 2′-5′-oligoadenylate synthetase 1, which is central to innate immune responses and interferon signaling. This suggests a potential role in immune-mediated colorectal carcinogenesis. In addition to its association with CRC and colon polyps, *OAS1* was also significantly associated in the multi-trait analysis of CRC and angina pectoris (Supplementary Table S5), highlighting a possible link between immune system regulation, chronic inflammation, and cardiovascular health. Given the well-established role of *OAS1* in antiviral defense mechanisms and immune modulation, its pleiotropic effects may extend beyond CRC to systemic inflammatory processes that contribute to both cancer and cardiovascular diseases. To illustrate the pleiotropic effect of rs2525858, we generated a LocusCompare plot (Figure 3), showing the colocalization of association signals between colorectal cancer and colon polyps. The high degree of overlap in association patterns, strong LD structure, and consistent effect estimates across traits indicate that *OAS1* may have a shared causal role in colorectal neoplasia and immune regulation. This strengthens the hypothesis that immune-related mechanisms contribute to CRC susceptibility and that *OAS1* may serve as a key genetic link between cancer and inflammatory pathways.

## 4 Discussion

In this study, we leveraged multi-trait GWAS to identify pleiotropic loci influencing colorectal cancer and genetically correlated traits, revealing novel associations that might have been missed in single-trait analyses. We identified three novel associations in East Asian populations: *BET1L* at 11p15.5 (rs12226698), *OAS1* at 12q24.13 (rs2525858), and *BMP2* at 20p12.3 (rs4813802). Among these, *BET1L* and *OAS1* were previously unreported in colorectal cancer studies, while *BMP2* had been identified in European populations, highlighting both novel and shared genetic risk factors across ancestries.

Colocalization analysis confirmed that the association signals at *BET1L* and *OAS1* were shared between colorectal cancer and colon polyps, supporting a common genetic basis for adenoma progression to malignancy. The strong correlation between association signals at *BET1L* suggests that this locus plays a role in early tumorigenesis, potentially through its involvement in Golgi-mediated vesicular transport and protein secretion, which are essential for maintaining epithelial homeostasis. The identification of *BET1L* and *OAS1* as novel loci associated with colorectal cancer (CRC) in East Asian populations provides significant insights into the disease’s pathogenesis. BET1L encodes a Golgi-associated membrane protein involved in vesicular transport. Recent studies have demonstrated that increased expression of *BET1L* promotes colorectal cancer cell growth both *in vitro* and *in vivo*, and is associated with advanced tumor stages and poorer patient prognosis. Mechanistically, *BET1L* has been linked to the steroid biosynthesis pathway through regulation of genes such as *HSD17B7*, *CYP27B1*, and *COMT*, suggesting its role in lipid metabolism and cellular stress responses in tumorigenesis. *OAS1* is a key component of the innate immune system, known for its role in antiviral defense by synthesizing 2′,5′-oligoadenylates that activate RNase L to degrade viral RNA. Beyond its antiviral functions, *OAS1* has been implicated in various cellular processes, including apoptosis and gene regulation. Notably, dysregulation of *OAS1* expression has been observed in several cancers, including colorectal cancer, where it may influence tumor progression by modulating the immune microenvironment. The association of *OAS1* with both colorectal cancer and cardiovascular diseases such as angina pectoris underscores the potential role of chronic inflammation and immune dysregulation as common mechanisms underlying these conditions. Collectively, these findings highlight the importance of metabolic and immune pathways in the pathogenesis of colorectal cancer and suggest that *BET1L* and *OAS1* could serve as potential biomarkers or therapeutic targets. Further research is warranted to elucidate the precise mechanisms by which these genes contribute to cancer development and their potential interplay with cardiovascular diseases.

Our findings contribute to the growing body of evidence that colorectal cancer (CRC) risk is influenced by pleiotropic genetic factors shared with related traits, particularly those involved in metabolic and immune regulation. Compared to previous GWAS, which primarily focused on single-trait analyses in European populations, our study highlights novel genetic loci specific to East Asians, emphasizing the importance of conducting genetic studies across diverse populations. These identified loci may have important clinical implications. Incorporating these variants into polygenic risk scores (PRS) could improve CRC risk prediction models in East Asian populations, enhancing the identification of high-risk individuals and supporting early screening and prevention strategies. Moreover, some of the identified loci may serve as potential therapeutic targets or biomarkers, informing personalized treatment approaches. However, several limitations should be considered. Although our analysis was based on large-scale summary statistics, our findings require further validation in independent cohorts and functional studies to confirm the causal variants and biological mechanisms at these loci. Additionally, while colocalization analysis provides strong evidence for shared genetic effects, fine-mapping studies are needed to pinpoint the specific causal variants driving these associations. Furthermore, we acknowledge that the lack of experimental validation is a limitation of our study, and future research should aim to address this gap.

Future research should focus on functional validation of *BET1L* and *OAS1*, as well as expanding multi-trait analyses to other genetically correlated conditions. Understanding the biological pathways influenced by these loci may provide insights into shared disease mechanisms and potential therapeutic targets. Given the role of *OAS1* in immune regulation, further investigation into its involvement in the tumor microenvironment could have implications for immunotherapy strategies. Similarly, exploring *BET1L*’s role in metabolic and vesicular transport pathways may uncover novel links between colorectal cancer and systemic metabolic health. Our study underscores the value of multi-trait GWAS in uncovering pleiotropic risk loci and advancing our understanding of colorectal cancer etiology in diverse populations.

## Data Availability

All GWAS summary statistics used in this study were obtained from publicly available datasets in the GWAS Catalog (https://www.ebi.ac.uk/gwas/).
