# Supplementary material for: Multi-trait GWAS identifies pleiotropic loci associated with colorectal cancer in East Asian populations
Source: Front Genet. 2025 Apr 15;16:1590652. doi: 10.3389/fgene.2025.1590652 (PMC12037559; doi:10.3389/fgene.2025.1590652)
Supplement: Supplementary file 2 [file DataSheet1.docx]

Supplementary Figure 1.

Supplementary Figure 1. Heat maps showing patterns of cell-type-specific enrichments of SNP-heritability for genetically associated traits across 396 cell-type-specific annotations. Each checkered rectangle reflects the z-score, scaled by traits. Red indicates enrichment, blue indicates depletion. Deeper color represents stronger magnitude of effects. Asterisks represent statistical significance withstanding multiple correction. The category of cell-types is color coded to the left. A) DNase (DNase I hypersensitive sites), B) H3K27ac, C) H3K36me3, D) H3K4me1, E) H3K4me3, and F) H3K9ac.

A) DNase

B) H3K27ac

C) H3K36me3

D) H3K4me1

E) H3K4me3

F) H3K9ac
